# CLINICAL VERSUS SUBJECTIVE PREDICTORS OF DISTRESS AND HEALTH SATISFACTION AND THE MODERATING ROLE OF PERCEIVED CONTROL

**DOI:** 10.1093/geroni/igad104.2532

**Published:** 2023-12-21

**Authors:** Gul Seckin

**Affiliations:** University of North Texas, Denton, Texas, United States

## Abstract

Coping with cancer in old age and a general decline in physical health may result in a lack of control over health. Cancer diagnosis tends to be associated with fear, uncertainty, and loss. It also challenges one’s assumptions about the sense of the self and bodily image. This paper explores health perceptions (distress with somatic symptoms, treatment side effects, and satisfaction with health status) in a sample (N=350) of older individuals who reported being treated with cancer (M age=50.23, S.D. =10.91). Three models of hierarchical multiple regressions were performed to estimate the impact of sociodemographic, medical, and psychosocial variables and perceived control over health on subjective health indicators. The results indicated that lower income was associated with greater distress with physical symptoms (Slope = -.12; B= -.17, p =.002), older age was associated with less distress with cancer treatment side effects (Slope = -.01; B= -.12, p =.019) and gender (female) were negatively associated with satisfaction with health status (Slope = -.36; B= -.15, p =.002). Even though the higher stage of cancer was a significant covariate for greater distress with somatic symptoms (Slope = .12; B= .17, p =.001), it was not a significant covariate for distress with treatment side effects and satisfaction with health status. Perceived control over health significantly moderated the association between research covariates and satisfaction with health. These findings suggest that assessing differential aspects of self-rated health may provide health professionals with important information to supplement their diagnosis to understand the patient’s illness experience better.

